# Literary Notes

**Published:** 1900-03

**Authors:** 


### LITERARY NOTES.

THE annual report of the Children's Hospital of Buffalo, N.Y., for the year ending October, 1899, as been received. The perusal of the report will afford pleasure to anyone interested in this beautiful children's charity. There are now eleven free beds in the hospital, and thirty-eight children, nearly one-third of the whole number cared for during the year, received free treatment. Only six deaths occurred, a remarkably low death-rate when one reads the list of diseases treated during the year.

---

THE New York *Lancet*, with which is incorporated the *Archives of Gynecology, Obstetrics and Pediatrics* has passed into the editorial management of Dr. Walter B. Chase, and his son, Dr. Carroll Chase. This must prove advantageous to the *Lancet*, as the editorial experience of Dr. Chase, Sr., and the energy of Dr. Chase, Jr., will infuse an activity into the journal that will interest its readers. We extend our cordial greetings to the editors with best wishes for their individual and journalistic prosperity.

THE *Regular Medical Visitor* is the name of a new medical periodical from St. Louis, Mo., the first number of which bears the date of January 15, 1900. It is edited by Dr. George Howard Thompson, who announces that the *Visitor* has "come to stay"—a circumstance that will be devoutly wished by all its St. Louis neighbors, we have no doubt. At all events we extend cordial New Year greetings to the *Visitor* with the hope that its fondest hopes may be realised.

---

THE *Stylus*, is the name of another new medical monthly from St. Louis, that has put in an appearance since the beginning of the year. It is edited by Dr. William Porter, who is a veteran journalist, assisted by Dr. Robert M. Ross. The first issue bears the date of February, 1900, and certainly bears the impress of experience and ability in its make-up and material. Our most cordial good wishes are presented to *The Stylus*.

---

DR. GEORGE F. BUTLER, of Chicago, formerly editor of *The Medical Standard*, has assumed editorial management of a new journal to be called *The Doctors' Magazine*. Dr. Butler is an author of repute, and we are publishing in this issue of the JOURNAL reviews of some of his recent books. We cannot doubt that his experience will assure success to his new enterpirise.

---

MESSRS. WILLIAM WOOD & CO. have the manuscripts of numbers of books submitted to them, which, while not calculated to find extensive sale to the medical profession generally, are of great interest and importance to specialists and practitioners working along the same lines. Their arrangements with foreign publishers also frequently include similar books. It is purposed to offer for sale very small editions of some of these, in certain cases not exceeding one hundred copies in all. After these are disposed of, they can no longer fill orders for them. Such books will be announced hereafter as "*Limited Edition*," and prompt application will be necessary to obtain copies.

This enterprising house also announces the publication of a new book entitled, Venereal diseases: their complications and sequelæ, by Edward L. Keyes, A. M., M. D., late professor of dermatology and genito-urinary surgery in the Bellevue Hospital Medical College; consulting surgeon to Bellevue Hospital, etc., etc., and Charles H. Chetwood, M. D., professor of genito-urinary surgery in the New

York Polyclinic College and Hospital; visiting surgeon to Bellevue Hospital, etc.

This book is divided into two parts: Part I., Acute and chronic urethritis; complications and sequelæ. Part II., chancroid and syphilis; embodying the senior author's original teaching concerning these diseases. A valuable chapter on nervous syphilis has been contributed by Dr. Pearce Bailey, consulting neurologist at St. Luke's Hospital.

It is an octavo volume, 364 pages, beautifully illustrated by eight full page plates, in black and colors, and one hundred and seven engravings. Bound in muslin. Price, $2.75 net.

---

THE *Mechanics of Surgery* is the title of a brochure issued by Charles Truax, of Chicago, head of the well-known instrument house of Truax, Greene & Co. The pamphlet contains excerpts from a book recently issued by Mr. Traux with the same title, as well as press and individual notices of the work. We printed an extended review of *The Mechanics of Surgery* in the issue of the JOURNAL for December, 1899, page 391, to which the attention of our readers is invited. The book is a unique and helpful presentation of and important adjunct to the work of the surgeon. Its price is $3.00 net.

